# Development and Validation of a Short Food Frequency Questionnaire to Measure Dietary Intake of a Selection of Immune-Modulating Nutrients in Patients with Established Peripheral Arterial Disease

**DOI:** 10.3390/nu13103316

**Published:** 2021-09-23

**Authors:** Bianca J. Collins, Christopher L. Delaney, Jade E. Boffo, Michelle D. Miller

**Affiliations:** 1 Nutrition and Dietetics, College of Nursing and Health Sciences, Flinders University, Bedford Park, SA 5042, Australia; bianca.collins@flinders.edu.au; 2 Department of Vascular Surgery, Flinders Medical Centre, Flinders University, Bedford Park, SA 5042, Australia; chris.delaney@sa.gov.au; 3 Caring Futures Institute, College of Nursing and Health Sciences, Flinders University, Bedford Park, SA 5042, Australia; jade.boffo@flinders.edu.au

**Keywords:** dietary intake, food frequency questionnaire, atherosclerosis, peripheral arterial disease

## Abstract

Nutrients with the ability to modulate the immune system (immune-modulating nutrients; IMN) may help prevent the development and progression of atherosclerosis, the main disease process underlying peripheral artery disease (PAD). Currently, no screening tool exists to measure IMN intake; therefore, the aim of this project is to develop and validate a short food frequency questionnaire (FFQ) that measures dietary intake of 14 nutrients with proposed immune-modulating effects, identified by the literature (copper, iron and zinc, vitamins A, C, D and E, alpha linolenic acid, total long-chain omega-3 fatty acids, arginine, glutamic acid, isoleucine, leucine and valine) in patients with established PAD. A 21-item FFQ was developed to measure average daily intake of IMNs over the past 12 months. Participants (*n* = 106) were recruited from Flinders Medical Centre, where they completed the FFQ followed by the reference method, a diet history reflecting usual intake over the past week. The mean age of participants was 72 years, with 83% being male (*n* = 88). Bland–Altman analysis resulted in a statistically non-significant *p*-value (*p*-value > 0.05) for 12 out of 14 nutrients, demonstrating good agreement between the two methods. Additionally, over 50% of nutrients had a sensitivity or specificity >70%. Consequently, the novel 21-item FFQ was determined to be a promising measure of dietary intake of 14 IMNs in patients with PAD when compared to the reference method of a diet history, and it is recommended that further investigations of the utility against biomarkers be explored in the future.

## 1. Introduction

Peripheral artery disease (PAD) is a local manifestation of the systemic disease state atherosclerosis, affecting over 200 million people globally [1,2,3,4,5]. Peripheral artery disease is associated with increased risk of non-fatal cardiovascular events, limb amputation and mortality, making it a major public health problem [1,5,6,7].

Atherosclerosis and subsequently PAD is initiated by endothelial cell dysfunction [2,3,4]. Normally, the endothelial cells (EC) lining the cardiovascular system contribute to vascular homeostasis through the production of nitric oxide (NO), which acts as a vasodilator, decreases platelet aggregation and blocks the expression of pro-inflammatory and adhesion molecules [2,3,8,9]. Risk factors such as hyperlipidaemia, smoking, hypertension and diabetes mellitus result in abnormalities in the production or bioavailability of NO, termed EC dysfunction [3,4]. These changes result in the adhesion and infiltration of monocytes and low-density lipoproteins (LDL) to the subendothelial space, initiating a complex pathological sequence resulting in the formation of an atherosclerotic plaque and a state of systemic pro-inflammation and oxidation [4,10].

Diet can be used in the primary and secondary prevention of cardiovascular disease (CVD) such as atherosclerosis, through the modification of risk factors, including hyperlipidaemia and hypertension [11,12]. Dietary patterns include the Heart Foundation Heart Healthy Eating Pattern, the Dietary Approach to Stop Hypertension (DASH) and the Mediterranean diet, which promote the consumption of fruit, vegetables, wholegrains, legumes, healthy fats such as nuts and seeds, low-fat dairy products and small amounts of lean meat [11,12]. More recently, nutrition therapy has focused on the ability of specific nutrients to regulate the immune system through their antioxidant and anti-inflammatory properties, termed immune-modulating nutrients (IMN) [2,13]. Table 1 provides a summary of some of these immune-modulating nutrients and their corresponding immune pathway/mechanism. These IMNs were selected given their proposed mechanism of action and relevance to atherosclerotic disease, specifically peripheral arterial disease.

Investigation into the nutritional status of patients with PAD indicates that large proportions have deficiencies or low dietary intake of some IMNs [17,18]. A study in America found that the proportion of the PAD population meeting vitamin A, C and E recommendations based on dietary intake was 43, 67 and 0%, respectively [17]. Additionally, an Australian-based study looking at micronutrient status from fasting blood samples found that 78.1% of participants with PAD were deficient in vitamin C, 58.1% in vitamin D and 50% in iron and zinc [18]. Thus, the development of a screening tool such as a food frequency questionnaire (FFQ), designed to measure dietary intake of IMNs for which there is Food Composition Database data, would be beneficial to identify PAD patients with low intake of measurable IMNs. Subsequently, those with low intake of selected IMNs could be identified to receive nutrition guidance to improve intake, which may result in a reduced pro-inflammatory response and thus better patient outcomes [2].

A FFQ examines specific nutrients over a selected period of time to retrospectively measure usual intake [19]. Unlike other dietary assessment methods such as a diet history or weighed food record, a FFQ is inexpensive and has low participant burden, usually taking 10–20 min to complete [19]. While biological markers can be used to assess micronutrients, they are expensive, invasive and may not accurately reflect dietary intake due to increased nutrient turn-over from disease processes [19,20,21,22].

Currently, there are a few FFQs that measure individual or a small group of IMNs, but none that encompass a comprehensive range of IMNs [23,24]. The Cancer Council Diet Questionnaire for Epidemiological Studies version 3.2 measures various vitamins, minerals and fatty acids, but it does not measure dietary amino acid or copper intake [25]. Similarly, a FFQ measuring antioxidants exists, but it does not measure anti-inflammatory nutrients [26]. Additionally, while the Mediterranean Diet Adherence Screener FFQ looks at adherence to a dietary pattern characterised by high intake of some IMNs, it does not measure the intake of specific nutrients [27]. Due to the synergistic effect of IMNs, it is important to measure multiple IMNs [2]. Therefore, the creation of a FFQ that measures multiple immune-modulating vitamins, minerals, fatty acids, and amino acids would be novel.

Consequently, the aim of this study was to develop and validate a FFQ that measures dietary intake of a range of IMNs in patients with established PAD. It is hypothesised that there will be adequate agreement on a population level between the novel FFQ and reference method, that is clinically meaningful, to validate the FFQ as an accurate measure of IMN intake in patients with PAD.

## 2. Materials and Methods

### 2.1. Study Design and Population

This validation study sought to compare the agreement of a novel FFQ in capturing IMN intake from the PAD population, compared to the reference method of a diet history. Participants were approached via convenience sampling from Flinders Medical Centre (FMC) (Adelaide, Australia). Recruitment occurred for FMC inpatient and outpatient podiatry and vascular clinics from August to November 2020. Patients were invited to participate in this study if they were 18 years or over with established PAD that had been diagnosed angiographically or via ankle-brachial index [6]. No exclusion criteria were applied for comorbidities, medical management of PAD or disease related conditions. Population characteristics such as age, living situation and comorbidities were obtained from medical records or verbally from participants. Ethics approval was obtained from the Southern Adelaide Clinical Human Research Ethics Committee (SAC HREC). Best practice guidelines for validating self-reported dietary assessment methods were used to inform the study design and reporting of results [28].

Eligible participants were approached by a member of the clinical team to identify if they would be interested in participating in research. The university-trained student dietitian (B.J.C) or accredited dietitian (J.E.B) subsequently explained this study and obtained written consent. Upon recruitment, the FFQ was administered, followed by the diet history reference method [20]. If individuals could not participate immediately but wished to take part, the FFQ and diet history were administered over the phone at a later time. No follow-up was required.

### 2.2. Food Frequency Questionnaire Development

This semi-quantitative FFQ was developed to assess average daily intake of a sub-set of IMNs over the past 12 months and included foods with high- or moderate-IMN content (Supplementary Materials). Immune-modulating nutrients identified by the literature and included in the Australian Food Composition Database Release 1 were measured in the FFQ. The IMNs included in the FFQ are copper, iron and zinc, vitamins A, C, D (cholecalciferol) and E, alpha linolenic acid (ALA), total LC n-3 FA, amino acids arginine and glutamic acid, and branch chain amino acids isoleucine, leucine and valine. Other IMNs, such as polyphenols and fermentable dietary fibres, were identified in the literature search, but are not in the Food Composition Database and therefore could not be included in the FFQ.

#### 2.2.1. Selecting Food Items

The Australian Food Composition Database Release 1 and previous literature were used to identify foods that were high in the relevant IMNs [23,24,25,26,29]. This information was compared to the Australian Health Survey: Nutrition First Results (AHS) which identified the proportion of persons consuming food items by age and gender [30]. Population age was defined as 51 years and over based on previous studies in the PAD population and the increasing prevalence of PAD with age [6,17,18]. Consequently, a list of foods high in IMNs, or with moderate amounts of IMNs commonly consumed by the population were included. Food items similar in type and IMN value were subsequently grouped, such as sweet potato and carrots, both root vegetables that are high in retinol equivalents (Supplementary Materials, question 10). This resulted in a 21-question FFQ.

#### 2.2.2. Piloting the FFQ

The FFQ was piloted by an accredited dietitian (J.E.B) in the same population group in 2019, as recommended by the literature [20]. This identified the FFQ as underestimating iron, zinc and beta-carotene (currently reported as retinol equivalents) and overestimating vitamin C, vitamin E and amino acids. Changes made to correct these findings included the addition of retinol-equivalent rich root vegetables, separating fruits into low and high vitamin C content and the inclusion of sub-questions, discussed below.

#### 2.2.3. FFQ Question Design

The semi-quantitative FFQ was designed to obtain information about portion of foods and frequency of consumption (Supplementary Materials). Portion was measured in servings with a description of what constitutes a serving, or in household measures such as cups, teaspoons, tablespoons, or slices. The first five questions were structured to elicit the portion size of each food consumed per day. The remaining questions were structure in two parts; asking how often a food item was consumed within a 7 day period, followed by the portion size consumed in a sitting. For grouped food items exhibiting within-group variability in nutrient composition, a sub-question was included. This prompted for the selection of the specific food type consumed within the group, such as wholegrain, wholemeal or white bread under the general term ‘bread’ (Supplementary Materials, question 1). If participants consumed multiple types of food within the group (e.g., white and wholegrain bread), it was assumed that participants consumed the food in equal amounts and the total number of servings consumed for the group was equally divided between the types of foods selected when entered in the nutrient database. Additionally, participants were asked if they were taking supplements, and to provide the nutrient type and dosage.

### 2.3. Reference Method

The reference method of a diet history, reflective of the previous week’s intake, was selected as a valid method of obtaining dietary information that represents usual intake [19]. A diet history requires one administration and is considered to have a medium level of burden on participants [19]. Multiple day 24 h recalls or weighed food records were deemed too burdensome for the population due to the associated time commitment, therefore were not selected [19]. Furthermore, a single 24 h recall may not represent usual intake [19]. A validated long FFQ could not be used as a reference method as no FFQ exists that measures all the relevant nutrients. Additionally, blood tests were not used due to their invasive and expensive nature [20].

### 2.4. Administration of FFQ and Reference Method

The FFQ was interviewer administered by a university trained student dietitian (B.J.C) or an accredited dietitian (J.E.B) [20]. The interviewer asked the participant each question on the FFQ, describing the foods included for grouped items and providing examples of what constitutes a serving. A clock or stopwatch was used to record time to complete the FFQ.

The dietitian that administered the FFQ for a participant also administered the diet history, obtaining information about usual food and drink intake over a 7 day period. Information was collected chronologically from the first to last food or drink item consumed of the day, recording usual intake and variations in intake such as weekend consumption. Details about quantity, frequency, and specific food information such as type of milk was also obtained. Carers or family members of participants were able to aid in completion of the FFQ and diet history.

### 2.5. Databases

The FFQ nutrient database was designed to calculate intake of the included IMNs per day using the frequency and portion size described by participants. Nutrient value per 100 g was obtained from the Australian Food Composition Database Release 1 [29]. Additionally, information on average food weight per serving was obtained from FoodWorks 10. This information was combined to calculate nutrient value per serving of food in the FFQ database.

The diet history database was a modified version of the Australian Food Composition Database Release 1, providing nutrient values per 100 g for 1534 foods [29]. When combined with food weight data from FoodWorks 10, the database calculated the amount of the included IMNs consumed per day. Where possible, the nutrient values of food items without amino acid and copper values were estimated based on the available nutrient information for similar foods.

### 2.6. Statistical Analysis

Analysis was conducted using IBM SPSS Statistics Version 25. Demographic characteristics were expressed as frequency (n) and percentage (%) or mean and standard deviation (SD) for visually normally distributed data. To assess agreement between the FFQ and diet history, Bland–Altman plots were used. The difference between the two tools (FFQ—diet history) was plotted against the mean of the two measures (FFQ + diet history/2). The mean difference (bias) was subsequently calculated to indicate the direction and magnitude of bias. The limits of agreement (LOA) were determined by the mean difference ± 1.96*SD. To calculate the LOA, a minimum sample size of 50 was required; however, a larger sample of 100 participants was preferable [20]. Linear regression was then used to determine proportional bias. T-statistic with a *p*-value > 0.05 indicated no statistical significance and therefore acceptance of the null hypothesis.

Nutritional adequacy of participant’s diet was assessed based on nutrient reference values (NRV) for Australia and New Zealand by age and gender [31]. Participants were categorised as having adequate or inadequate intake based on the results from the FFQ and diet history, independently, expressed as percentage of participants with inadequate intake. Additionally, sensitivity and specificity were assessed to indicate the validity of the FFQ as a screening tool. Sensitivity was calculated by the number of true positives (inadequate intake) divided by the total number of positive results, and specificity was calculated by the number of true negatives (adequate intake) divided by the total number of negative results, based on the diet history as the reference method.

### 2.7. Clinical Significance

To determine if the FFQ was a valid measure of intake of the selected IMNs, the Bland–Altman analysis needed to be clinically meaningful, determined by the clinical context [32]. As no literature could be identified to determine a clinically meaningful cut-off value, the clinical judgement of an experienced accredited dietitian (M.D.M) was used. This was predetermined to be less than or equal to ½ a serving of high-IMN food, or 1 serving of moderate-IMN food. Similarly, the LOA were defined as clinically meaningful at less than or equal 2 or 4 servings of high- or moderate-IMN foods, respectively. High- and moderate-IMN foods were, respectively, determined by a nutrient composition greater than or equal to 25 and 10% of the Recommended Dietary Intake (RDI) per serving of food based on Food Standards Australia and New Zealand (FSANZ) [31,33]. Food servings high in IMNs were then extracted and their IMN value per serving was averaged for each nutrient to provide a basis for cut off values.

## 3. Results

### 3.1. Population Characteristics

From the 119 eligible participants approached, 108 consented to participate. Two withdrawals occurred, resulting in 106 participants completing the FFQ and diet history. Participant characteristics can be found in Table 1. Overall, there was a higher proportion of males compared to females who completed this study, with 83% being male. The mean age of participants was 72 with the oldest participant being 96 years old. The majority of participants lived at home, with six in residential aged care and one staying in temporary accommodation while undergoing chemotherapy. Additionally, 11 received meal delivery services. Ninety-five of the 106 participants were sampled from FMC outpatient services, of which, two participants completed the interview by phone. The average time to administer the FFQ was eight minutes, ranging from four to 20 min.

### 3.2. Agreement between FFQ and Diet History

**Figure 1:**
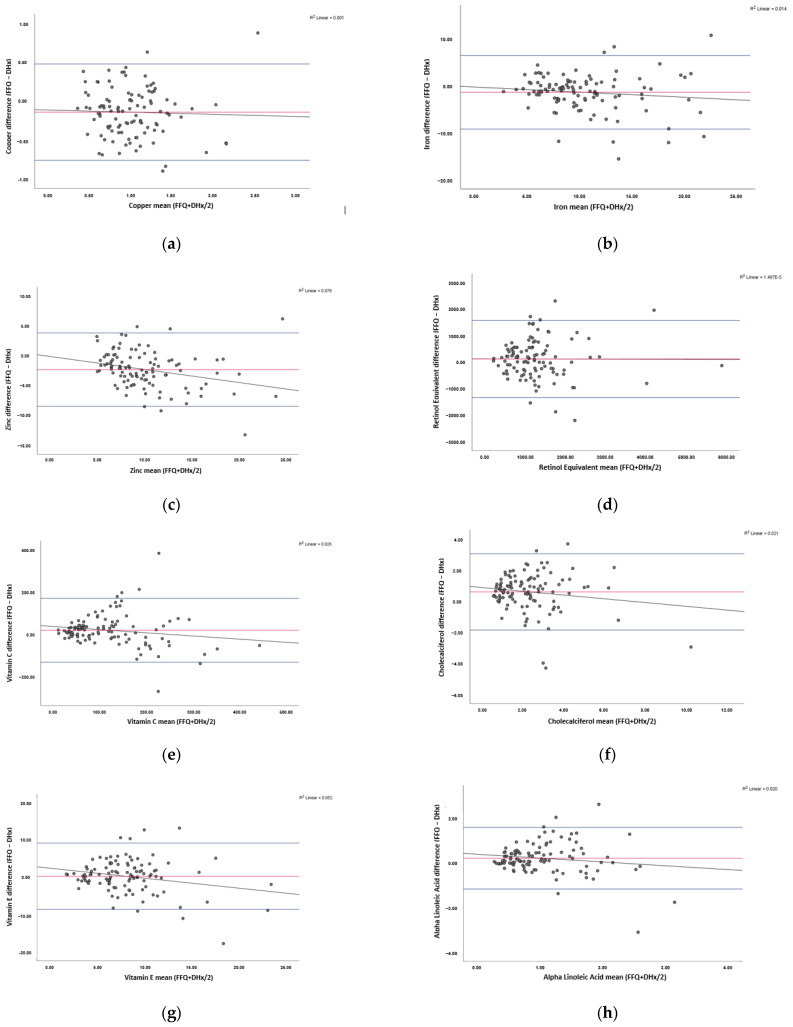

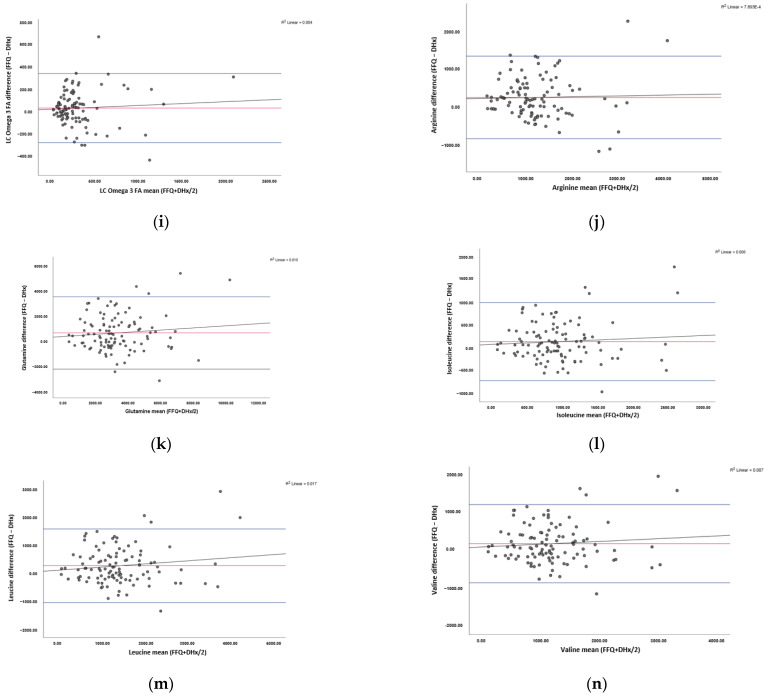
Bland–Altman plots showing the agreement between the food frequency questionnaire (FFQ) and diet history (DHx) in assessing IMN intake. Results shown for: (**a**) copper, (**b**) iron, (**c**) zinc, (**d**) vitamin A, (**e**) vitamin C, (**f**) vitamin D, (**g**) vitamin E, (**h**) alpha linolenic acid (ALA), (**i**) total long-chain omega-3 fatty acids (LC n-3 FA), (**j**) arginine, (**k**) glutamine, (**l**) isoleucine, (**m**) leucine, and (**n**) valine. The mean difference between the FFQ and DHx (bias) is depicted in red, limits of agreement where 95% of data points will lie are in blue, and linear regression is shown in black. *n* = 106.4.

Figure 1 illustrates the Bland–Altman plots, demonstrating the level of agreement between the FFQ and diet history. Visually, all plots have a good spread of points above and below the bias line. A summary of the bias, LOA, clinically acceptable ranges and proportional bias for all nutrients assessed are shown in Table 2.

For all minerals assessed, a negative bias was observed, whereas the remaining nutrients obtained a positive bias. For zinc, the bias was marginally outside the predefined clinically acceptable range of ±2.10 at −2.33 mg. Conversely, the bias for the remaining nutrients was within the clinically acceptable range. Similarly, the *p*-value was statistically non-significant (*p*-value > 0.05) for all nutrients except zinc and vitamin E.

The upper and lower LOA were outside of the predefined range for vitamin C. Furthermore, the lower LOA were outside the acceptable range for iron and zinc. Alternatively, copper, retinol, cholecalciferol, vitamin E, ALA, total LC n-3 FA and all amino acids had a clinically narrow LOA.

The T-statistic indicates that there is statistically significant proportional bias for zinc and vitamin E. For both nutrients, the linear regression seen in Figure 1 shows that as intake increases, the FFQ is more likely to underestimate intake. For the remaining nutrients, proportional bias was not statistically significant.

Additionally, of those that took supplements, all were able to describe the nutrient consumed, but were unable to specify the dosage taken for the FFQ and diet history.

### 3.3. Nutritional Adequacy of Diet

Average nutrient intake based on the FFQ and diet history are displayed in Table 3 with the NRV. Based on the validated diet history assessment, large proportions of participants were not meeting requirements for copper, zinc, cholecalciferol, vitamin E and ALA (Table 3). Additionally, a smaller proportion of participants were not meeting requirements for iron, vitamin A, vitamin C and total LC n-3 FA (Table 3).

Sensitivity and specificity of the FFQ are summarised in Table 4. Notably, copper, iron, zinc, vitamin D and vitamin E had a sensitivity greater than 75%. Alternatively, iron, retinol equivalents, vitamin C and LC n-3 FA had a specificity greater than 70%.

This study set out to develop a nutritional screening tool, in the form of a short FFQ, that can be used to identify PAD patients with inadequate dietary intake of selected IMNs. The potential beneficial effects of IMNs in reducing the development and progression of PAD, combined with the low dietary intake and micronutrient status of IMNs in PAD patients make this an important tool to develop [2,17,18]. This is the first FFQ that we are aware of that assesses dietary intake of IMNs as a group. This 21-item FFQ showed strong agreement with the diet history for copper, iron, vitamins A, C, E and D, ALA, total LC n-3 FA, arginine, glutamic acid, isoleucine, leucine and valine. Additionally, it showed moderate agreement with zinc. Consequently, this novel FFQ is a promising measure of dietary intake of these IMNs in the PAD population.

### 3.4. Agreement between the FFQ and Reference Method

Bland–Altman analysis showed adequate agreement between the FFQ and reference method of a diet history on a population level for all nutrients. These results were expected due to the use of previous FFQ validation studies and systematic reviews, which informed the methodology, structure and foods used in the validation of the IMN-FFQ [20,24,25,26,34]. The 21-item FFQ is shorter than other FFQs of interest, which contain 37 to 92 items [25,26]. Additionally, the use of a 297-item questionnaire is present in the literature [35]. The creation of a short FFQ that can accurately assess multiple nutrients is beneficial due to the reduced time for administration, therefore reducing participant burden.

For zinc, the FFQ underestimates dietary intake compared to the diet history. Additionally, proportional bias is present, which indicates the FFQ is more likely to underestimate intake as dietary intake increases. These results are contrary to the overestimate of zinc intake in a 74-item zinc-specific FFQ [23]. Subsequent analysis showed increasingly shorter versions of the zinc FFQ were more likely to underestimate intake, which is consistent with the literature [20,23]. This is particularly relevant to the IMN-FFQ as it contains 21-grouped food items and assesses 14 different nutrients, suggesting there might be food items missing that have moderate to high amounts of zinc. Despite underestimating zinc intake and showing proportional bias, zinc had a high sensitivity rating at 96.05%, therefore was able to accurately identify participants with inadequate intake. Additionally, zinc was only slightly out of the clinical range identified. This range was a novel way of assessing clinical meaning due to the inability to find existing methods in the literature. Considering the FFQ is intended for screening inadequate nutrient intake, the high sensitivity rating combined with a bias of –2.33 mg compared to the clinically significant value of ±2.10 mg, the FFQ may be considered a valid measure of dietary zinc intake in this population.

Limits of agreement define where 95% of the data points will lie and help to determine how well two methods agree on an individual level [32]. A smaller range, determined by the clinical context, corresponds to better agreement [32]. For vitamin C, the LOA were considered wider than the pre-defined clinically acceptable range of 2 servings of high vitamin C food. Discrepancies between the two methods could be due to difference in time periods captured. While a diet history is likely to capture usual intake, it only records intake over a 7 day period, compared to the FFQ which measures average intake over a year [19]. Dietary data obtained in the diet history may not account for seasonal variations in intake that the FFQ captures. This is a key consideration to make especially when assessing vitamin intake, due to the availability of these nutrients between seasons [34]. Previous studies have also observed wide LOA for vitamin C. In a study of 238 participants, the LOA ranged from −121.81 to 178.89 mg, with a bias of 28.54 mg [36]. Another study reported LOA of −110 to 120 mg, with a bias of 5 mg [37]. However, this second study only had 40 participants which may not be sufficient to accurately calculate the LOA [20]. Interestingly, despite the clinically wide LOA in the present study and literature, all had adequate agreement on a population level [36,37]. This indicates that the wide LOA obtained in the current study are not abnormal, therefore changes to the FFQ are not required at this stage.

The proportional bias for vitamin E indicates the FFQ is more likely to underestimate intake of vitamin E as intake increases. Interestingly, no other FFQ that reported proportional bias for vitamin E could be identified in the literature. All foods identified by the literature as high in vitamin E were included in the FFQ such as the fats of meat, poultry and fish, and smaller amounts in cereal and dairy foods, therefore the proportional bias is not likely due to missing food items [31]. Despite the proportional bias present, the FFQ is intended to be used as a screening tool for low intakes of IMNs. Vitamin E had a sensitivity of 75%, which indicates a good ability to detect inadequate intake. Furthermore, the proportion of participants with inadequate intake identified by the FFQ was 68%, compared to the 69.81% identified by the diet history. This highlights that despite the proportional bias, the FFQ can still be used to identify inadequate intake.

### 3.5. Selectivity and Specificity

As shown in Table 4, the sensitivity and specificity analysis presented some issues. A small proportion of participants had inadequate intake for retinol, vitamin C and total LC n-3 FA. These nutrients received a high specificity, but low sensitivity. Similarly, the reverse is true for nutrients that had large proportions of participants with inadequate intake, resulting in a high sensitivity and low specificity. These results could be impacted by the uneven distribution of participants with or without inadequate intake for each nutrient. Alternatively, when comparing average nutrient intake and the proportion of participants with inadequate intake based on the FFQ and diet history, results were similar between methods. Consequently, despite the poor sensitivity and specificity rating for some nutrients, the FFQ was still able to identify similar rates of inadequate intake and similar absolute values, when compared to the diet history.

In the present study, a large proportion of participants’ dietary intake were inadequate for copper, zinc, vitamin D, vitamin E and ALA. An American study looking at the dietary intake in the PAD population identified 57, 33 and 100% of participants did not meet requirements for vitamin A, C and E, respectively [17]. Contrary to this, an Australian study looking at micronutrient status of PAD patients found that 16.7% were deficient in vitamin A, 78.1% in vitamin C, 0% in vitamin E, 58.1% in vitamin D and 50% in iron and zinc [18]. This variation highlights the importance of the screening IMN intake and subsequent dietary intervention in this patient group.

### 3.6. Supplementation

Supplement use is important to identify in dietary collection methods, with nearly one-third of Australians reported consuming dietary supplements in the AHS [20,30,34]. In the present study, participants who consumed dietary supplements were unable to describe the dosage consumed. According to the literature, to accurately determine nutrient intake from supplements, information about the nutrient type and dosage is required [20,34]. This is highlighted by the variability in nutrients shown in the FSANZ dietary supplement nutrient database which contains the nutrient content of the 2163 types of supplements collected from the AHS [38]. A study by Satia et al. [26] utilised detailed prompting questions about supplement use to elicit dosage information. While this was not valid in the self-administered FFQ, it could be beneficial in an interview administered FFQ [26].

Previous studies have found that gender ratios range from majority males to majority females in more severe disease progression of PAD [6]. While the larger number of males to females recruited in this study could be due to convenience sampling, there is no clear expected gender ratio in this population group. Consequently, the result from this study can likely be applied to the wider PAD population.

### 3.7. Limitations

An important limitation to consider is that the FFQ only captures the IMNs where nutrient data were available in the selected nutrient composition database. Hence, there are other IMNs known to exist, but accurate measure of intake was not possible given the data collection methodology and electronic software used in this study. For example, polyphenols are known to regulate immune response through activation of signaling pathways and induction of epigenetic changes in intestinal mucosa [39]. Likewise, short chain fatty acids produced from the digestion of fermentable dietary fibre increases the cytotoxic activity of cells important in immune response [40]. Although the FFQ does not measure these important IMNs, the nutrients examined have particular relevance to the population studied.

A key limitation of the FFQ is the lack of Australian amino acid information available for food types in the Australian Food Composition Database Release 1 [29]. This database was chosen as it contained information for most of the relevant nutrients [29]. However, amino acid data were not available for high-protein food items including chicken, red meat, some seafood and cooked eggs. A study in Poland highlighted that meat and meat products contributed 30.9–46.1% of arginine, glutamic acid, leucine, isoleucine and valine to total dietary amino acid intake. Thus, it was important to estimate amino acid quantities in these food items based on similar food types. For example, the amino acid content of canned salmon was used to estimate the amino acid value of a fillet of salmon. Nutrient databases from other countries were not used to gain this information due to limited access, different food systems, and difficulty matching up foods in the databases. Notably, this limitation would not affect the validation of the FFQ, as the FFQ and diet history used nutrient information from the same database. However, dietary intake data obtained for amino acids is not a true representation of actual amino acid intake. Despite this, it remained important to include amino acids in the FFQ.

A further limitation is the potential bias that may be present due to the same interviewer administering the FFQ and diet history sequentially to the one participant. The potential bias arises from the interviewer wanting the FFQ to be validated against the reference method, which is administered directly after the FFQ. However, care was taken to ensure the interviewer only recorded intake described by the participant for each method. Although this process of administration causes potential bias in the results, the methodology was informed by the literature. Cade et al. [20] indicates that FFQ should be administered prior to the reference method to reduce participant-awareness about dietary intake. Additionally, the potential use of different interviewers administering each dietary assessment method presents the additional problem of the results being affected by differences in portion estimation between interviewers. Despite the benefits of a diet history, such as providing a representation of usual intake over the past 7 days and requiring only one administration period, it is limited by the skill of the interviewer in prompting for food intake data and estimation of portion size, and is subject to observer and recall bias [19]. Alternatively, a weighed food record prospectively records dietary intake, but it possesses known limitations such as participant and researcher burden [19]. Additionally, blood samples provide an objective measure which does not rely on participant’s memory; however, these are seen to be invasive and expensive [20]. Furthermore, the relationship between dietary intake and biomarker levels is not always clear and can be impacted by nutrient uptake, utilisation, metabolism and excretion, and therefore may not reflect dietary intake and therefore dietary changes that can be made [20]. However, this study could be strengthened through validation of the FFQ against biomarkers to reduce potential interviewer bias.

### 3.8. Strengths

The major strength of this study was the creation of a novel FFQ that is specific to the testing population. Australian food and nutrient intake data were constantly referenced throughout the development of the questionnaire. This ensured the FFQ was specific to dietary intake patterns of Australians in the age group of interest. Additionally, the most up to date Australian nutrient database was used, making the FFQ database specific to the Australian food supply. These methods could be used to adapt the FFQ to various cultures. Evidence-based practice was applied through each step of the development, administration and validation of the FFQ.

Further strengths lie in the rigorous pre-testing that was conducted in the same population group to identify flaws within the FFQ that could be amended to better the tool and make it more specific to the population’s dietary patterns. Furthermore, the FFQ included 21 items and took only 8 min to complete, while assessing dietary intake of 14 different nutrients.

### 3.9. Implications for Practice

In practice, the FFQ could be used for the screening of PAD patients on a population level to identify those with low dietary intake of IMNs. Patients with low intake could then receive nutritional therapy to amend their intake, which may result in a reduced pro-inflammatory response [2]. Consequently, patients may have better outcomes, thus reducing the risk of non-fatal cardiovascular events, limb amputation and mortality [1,2,5,6,7].

It has been noted that nutritional therapy should be specific to the patient’s individual situation [2]. Therefore, the screening and identification of specific nutrients that are low in intake is an important step in individualising nutrition care in this area.

## 4. Conclusions

This novel FFQ demonstrated clinically meaningful agreement between the FFQ and diet history reference method on a population level for copper, iron, vitamin A, C, D and E, ALA, total LC n-3 FA, arginine, glutamine, isoleucine, leucine and valine. Despite zinc being outside of the clinically meaningful range, it provided a high sensitivity rating, indicating that it is accurate at identifying participants with inadequate intake. Consequently, the FFQ may be deemed a valid measure of dietary IMN intake in patients with established PAD although validation against biomarkers could strengthen this position.

## Figures and Tables

**Table 1:** Immune pathway and mechanism of selected immune-modulating nutrients.

Immune-Modulating Nutrient	Immune Pathway/Mechanism
Glutamine	Modulates immune system and suppresses acute inflammatory responses when administered to gastrointestinal surgical patients, reducing infectious complications and length of stay [14,15], highlighting potential in PAD population to impede the development and progression of atherosclerosis through anti-inflammatory and antioxidant properties, mitigating the pro-inflammatory and oxidative state of atherosclerosis [2].
Arginine	
Nucleotides	
Omega-3 fatty acids (LC n-3 FA)	
Vitamin E	Reduces suppression of adhesion molecules resulting in reduced recruitment of monocytes [9]
Vitamin C	Increases endothelial cell production of NO, thus blocking pro-inflammatory and adhesion molecules [9,16].
B-vitamins	
L-arginine	
Vitamin D	Decreases levels and uptake of LDL which contribute to inflammation and disease progression [8,9,13].
LC n-3 FA	
Zinc	Decreases inflammation through modulating the release of pro-inflammatory cytokines [8,9,13].

**Table 2:** Characteristics of the participants in the validation of immune-modulating nutrients food frequency questionnaire against a diet history.

Characteristics	Mean ± SD or *n* (%)
Age (years)	72 ± 11.49
Gender	
Male	88 (83)
Female	18 (17)
Living situation	
Home	99 (93.4)
Residential Aged Care	6 (5.7)
Other	1 (0.9)
Recruitment location	
Inpatient	11 (10.4)
Outpatient	95 (89.6)
Time for FFQ (minutes)	8.25 ± 2.68

*n* = 106. Abbreviations: SD, standard deviation; n, number of participants.

**Table 3:** Summary of results from the Bland–Altman analysis comparing the food frequency questionnaire to diet history, including the bias, upper and lower limits of agreement (LOA), clinically acceptable ranges for the bias and LOA, the T-statistic and the corresponding *p*-value.

Nutrient	Bias	Upper LOA	Lower LOA	Clinically Acceptable Bias (±) ^a^	Clinically Acceptable LOA (±) ^b^	T-Statistic	*p*-Value ^c^
Copper (mg)	−0.14	0.47	−0.75	0.28	1.10	−0.35	0.73
Iron (mg)	−1.53	6.24	−9.29	2.04	8.17	−1.22	0.23
Zinc (mg)	−2.33	3.76	−8.42	2.10	8.39	−2.96	0.00
Retinol Equivalents (µg)	115.94	1566.58	−1334.69	1197.99	4791.95	−0.04	0.97
Vitamin C (mg)	16.06	166.50	−134.39	24.38	97.54	−1.66	0.10
Cholecalciferol (D3) (µg)	0.58	3.01	−1.86	2.70	10.79	−1.49	0.14
Vitamin E (mg)	0.34	9.19	−8.51	2.75	10.98	−2.39	0.02
Alpha Linolenic Acid (g)	0.17	1.52	−1.19	0.27	1.07	−1.47	0.15
Total LC n-3 FA (mg)	29.32	337.09	−278.46	542.96	2171.83	0.65	0.52
Arginine (mg)	241.96	1329.48	−845.55	316.90	1267.62	0.29	0.78
Glutamic Acid (mg)	564.67	3417.36	−2288.03	687.80	2751.19	1.02	0.31
Isoleucine (mg)	145.55	995.88	−704.79	203.51	814.04	0.78	0.44
Leucine (mg)	261.33	1563.47	−1040.80	312.40	1249.60	1.34	0.48
Valine (mg)	182.90	1217.14	−851.33	243.73	974.92	0.89	0.38

^a^ Based on ½ or 1 serving of foods with a high or moderate nutrient composition per serving, respectively. ^b^ Based on 2 or 4 servings of foods with a high or moderate nutrient composition per serving, respectively. ^c^ Statistically significant *p*-value at ≤0.05. *n* = 106.

**Table 4:** Average dietary intake of nutrients based on the food frequency questionnaire (FFQ) and diet history, and the upper nutrient reference values (NRV); and proportion of participants with inadequate dietary intake of nutrients compared to the nutrient reference values based on the food frequency questionnaire (FFQ) and diet history, and the sensitivity and specificity of the FFQ in measuring inadequate dietary intake of nutrients when compared to the diet history.

		Average Nutrient Intake		Proportion Inadequate Intake (%) ^‡^		Sensitivity and Specificity	
Nutrient	NRV ^a^	FFQ	Diet History	FFQ	Diet History	Sensitivity (%) ^b^	Specificity (%) ^c^
Copper (mg)	1.7	0.94	1.08	94.34	89.62	98.95	45.45
Iron (mg)	8	9.69	11.22	43.40	26.42	89.29	73.08
Zinc (mg)	14	8.94	11.27	86.79	71.70	96.05	36.67
Retinol Equivalents (µg)	900	1339.56	1223.61	33.02	41.51	50.00	79.03
Vitamin C (mg)	45	128.49	112.43	17.92	27.36	48.28	94.81
Cholecalciferol (D3) (µg)	15	2.53	1.95	100.00	100.00	100.00	-
Vitamin E (mg)	10	8.30	7.96	68.00	69.81	75.68	50.00
Alpha Linolenic Acid (g)	1.3	1.12	0.95	66.04	75.47	67.65	34.72
Total LC n-3 FA (mg)	160	325.63	296.31	24.53	32.08	58.82	91.67
Arginine (mg)	NA	1365.69	1123.73				
Glutamic Acid (mg)	NA	3578.00	3013.33				
Isoleucine (mg)	NA	1029.00	883.45				
Leucine (mg)	NA	1601.91	1340.58				
Valine (mg)	NA	1276.19	1093.29				

^a^ Based on the upper NRV for males or females aged 51 or older. *n* = 106. Abbreviations: NRV, nutrient reference values; NA, not available; total LC n-3 FA, total long-chain omega-3 fatty acids. ^‡^ Based on the nutrient reference values specific to each participant’s age and gender. ^b^ Calculated by number of true positives (inadequate intake) divided by the total number of positive results, based on the diet history as the reference method. ^c^ Calculated by the number of true negatives (adequate intake) divided by the total number of negative results‡, based on the diet history as the reference method. *n* = 106. Abbreviations: total LC n-3 FA, total long-chain omega-3 fatty acids.

## Supplementary Materials

The following are available online at https://www.mdpi.com/article/10.3390/nu13103316/s1, Supplementary file: Diet Questionnaire.
