# Supplementary material for: Development and Validation of a Short Food Frequency Questionnaire to Measure Dietary Intake of a Selection of Immune-Modulating Nutrients in Patients with Established Peripheral Arterial Disease
Source: Nutrients. 2021 Sep 23;13(10):3316. doi: 10.3390/nu13103316 (PMC8540050; doi:10.3390/nu13103316)
Supplement: Supplementary file 1 [file nutrients-13-03316-s001.zip › nutrients-1339885-supplementary.pdf]

Diet Questionnaire

This questionnaire asks about the types of food you have eaten in the last 12 months.
This includes all foods and drinks you have eaten for meals, in-between meals, and away from home.
Every question should be answered, even if it is just an estimate.

Over the last <u>12 months</u> how much of the following foods did you eat <u>on average each day</u>?

1. Bread (1 slice bread = ½ bread roll)

☐ None ☐ ½ slice ☐ 1 slice ☐ 2 slices ☐ 3 slices ☐ 4 slices ☐ 5 slices ☐ 6 slices

What type of bread?

☐ Whole grain ☐ Wholemeal ☐ White

2. Milk, including on its own as a beverage, with cereal, and as an ingredient to drinks such as tea or coffee (1 cup = 250ml)

☐ None ☐ ½ cup ☐ 1 cup ☐ 1½ cup ☐ 2 cups ☐ 2½ cups ☐ 3 cups ☐ 4 cups

What type of milk?

☐ Full fat ☐ Reduced fat ☐ Skim

3. Margarine (Flora, Nuttelex, MeadowLea, Olive Grove) including spread on bread or toast, and on vegetables (1 teaspoon (thin spread) = 5g; 4 teaspoons = 20g = 1 tablespoon (thick spread))

☐ None ☐ 1 tsp ☐ 2 tsp ☐ 3 tsp ☐ 1 Tbs ☐ 2 Tbs ☐ 3 Tbs ☐ 4 Tbs

What type of margarine?

☐ Flora or Nuttelex ☐ MeadowLea ☐ Olive Grove

4. Oil in cooking, on vegetables or salad, or on bread or toast (1 teaspoon = 5g; 4 teaspoons = 20g = 1 tablespoon) *Please estimate the amount that was in your meal alone.*

☐ None ☐ 1 tsp ☐ 2 tsp ☐ 3 tsp ☐ 1 Tbs ☐ 2 Tbs ☐ 3 Tbs

What type of oil?

☐ Sunflower oil ☐ Canola or vegetable oil ☐ Olive oil

5. Fruit

5.1 Apple, pear, banana, grapes (1 serve = 1 whole medium apple, pear, banana or 1 cup grapes or 2/3 cup tinned fruit)

☐ None ☐ ¼ serve or 1 serve 2/wk ☐ ½ serve or 1 serve 3-4/wk ☐ 1 serve ☐ 2 serves ☐ 3 serves ☐ 4 serves ☐ 5 serves

5.2. Orange, mandarin, kiwi fruit, berries (1 serve = 1 whole orange or 2 small mandarins / kiwi fruit or 1 cup berries)

☐ None ☐ ¼ serve or 1 serve 2/wk ☐ ½ serve or 1 serve 3-4/wk ☐ 1 serve ☐ 2 serves ☐ 3 serves ☐ 4 serves ☐ 5 serves

Please answer the following questions for each food type:

*a) Over the last 12 months, on average **how often** did you eat this food **in a week**?*
*b) Over the last 12 months, on average **how much** of the following foods did you eat **in a single sitting**?*
If you answer 'never' to a), please skip to the next section.

6. Juice

a) Times per week

☐ Never ☐ 1/week ☐ 2/week ☐ 3/week ☐ 4/week ☐ 5/week ☐ 6/week ☐ 7/week

b) Portion in one sitting (1 cup = 200mL)

☐ ¼ cup ☐ ½ cup ☐ 1 cup ☐ 1½ cups ☐ 2 cups ☐ 3 cups ☐ 4 cups

7. Breakfast cereal

a) Times per week

☐ Never ☐ 1/week ☐ 2/week ☐ 3/week ☐ 4/week ☐ 5/week ☐ 6/week ☐ 7/week

What type of cereal?

☐ Oats, porridge, or muesli ☐ Other (rice-, corn-, bran-, wheat-based cereals)

b) Portion in one sitting (1 serve = 1 cup flaked cereal or 2 Weet-Bix™ or ½ cup oats or muesli)

☐ ½ serve ☐ 1 serve ☐ 2 serves ☐ 3 serves ☐ 4 serves ☐ 5 serves ☐ 6 serves

8. Rice, pasta, or noodles

a) Times per week

☐ Never ☐ 1/week ☐ 2/week ☐ 3/week ☐ 4/week ☐ 5/week ☐ 6/week ☐ 7/week

b) Portion in one sitting (½ cup = 1 small handful)

☐ ¼ cup ☐ ½ cup ☐ 1 cup ☐ 1½ cups ☐ 2 cups ☐ 2½ cups ☐ 3 cups

9. Potato

a) Times per week

☐ Never ☐ 1/week ☐ 2/week ☐ 3/week ☐ 4/week ☐ 5/week ☐ 6/week ☐ 7/week

b) Portion in one sitting (1 serve = ½ medium potato or ¼ cup mash)

☐ ½ serve ☐ 1 serve ☐ 2 serves ☐ 3 serves ☐ 4 serves ☐ 5 serves ☐ 6 serves

10. Sweet potato or carrot

a) Times per week

☐ Never ☐ 1/week ☐ 2/week ☐ 3/week ☐ 4/week ☐ 5/week ☐ 6/week ☐ 7/week

b) Portion in one sitting (1 serve = 1 large carrot or ½ medium sweet potato)

☐ ¼ serve ☐ ½ serve ☐ 1 serve ☐ 2 serves ☐ 3 serves ☐ 4 serves ☐ 5 serves

11. Tomato and tomato sauces e.g. pasta sauce

a) Times per week

☐ Never ☐ 1/week ☐ 2/week ☐ 3/week ☐ 4/week ☐ 5/week ☐ 6/week ☐ 7/week

b) Portion in one sitting (1 serve = 120g = 1 medium tomato or ½ cup tomato pasta sauce)

☐ ¼ serve ☐ ½ serve ☐ 1 serve ☐ 2 serves ☐ 3 serves ☐ 4 serves ☐ 5 serves

12. Leafy green vegetables e.g. lettuce, spinach, silver beet

a) Times per week

☐ Never ☐ 1/week ☐ 2/week ☐ 3/week ☐ 4/week ☐ 5/week ☐ 6/week ☐ 7/week

b) Portion in one sitting (1 serve = 1 cup raw or ½ cup cooked)

☐ ¼ serve ☐ ½ serve ☐ 1 serve ☐ 2 serves ☐ 3 serves ☐ 4 serves ☐ 5 serves

13. Green peas – from fresh, frozen or canned

a) Times per week

☐ Never ☐ 1/week ☐ 2/week ☐ 3/week ☐ 4/week ☐ 5/week ☐ 6/week ☐ 7/week

b) Portion in one sitting (½ cup = 1 small handful)

☐ 2 Tbs ☐ ¼ cup ☐ ½ cup ☐ 1 cup ☐ 2 cup

14. Cruciferous vegetables e.g. cauliflower, cabbage, broccoli, Brussel sprouts, Bok choy

a) Times per week

☐ Never ☐ 1/week ☐ 2/week ☐ 3/week ☐ 4/week ☐ 5/week ☐ 6/week ☐ 7/week

b) Portion in one sitting (1 serve = 1 cup raw or ½ cup cooked)

☐ ¼ serve ☐ ½ serve ☐ 1 serve ☐ 2 serves ☐ 3 serves ☐ 4 serves ☐ 5 serves

15. Beef, veal, lamb, or pork, including as roast, steak, mince, chops, stew etc.

a) Times per week

☐ Never ☐ 1/week ☐ 2/week ☐ 3/week ☐ 4/week ☐ 5/week ☐ 6/week ☐ 7/week

b) Portion in one sitting (1 serve = 65g = ½ cup mince or 1 chop or 1 slice roast meat or 1 sausage)

☐ ½ serve ☐ 1 serve ☐ 2 serves ☐ 3 serves ☐ 4 serves ☐ 5 serves

16. Chicken or other poultry

a) Times per week

☐ Never ☐ 1/week ☐ 2/week ☐ 3/week ☐ 4/week ☐ 5/week ☐ 6/week ☐ 7/week

b) Portion in one sitting (1 serve = 80g = ½ breast fillet or 1 chicken drumstick or 2/3 chicken thigh)

☐ ½ serve ☐ 1 serve ☐ 2 serves ☐ 3 serves ☐ 4 serves ☐ 5 serves ☐ 6 serves

17. Oily fish e.g. salmon, tuna, sardines, mackerel

a) Times per week

☐ Never ☐ 1/week ☐ 2/week ☐ 3/week ☐ 4/week ☐ 5/week ☐ 6/week ☐ 7/week

b) Portion in one sitting (1 serve = 100g = 1 fillet or 1 small can)

☐ ½ serve ☐ 1 serve ☐ 2 serves ☐ 3 serves ☐ 4 serves ☐ 5 serves ☐ 6 serves

What type of fish?

☐ Salmon ☐ Tuna ☐ Sardines ☐ Mackerel

18. Other white fish e.g. whiting, flathead

a) Times per week

☐ Never ☐ 1/week ☐ 2/week ☐ 3/week ☐ 4/week ☐ 5/week ☐ 6/week ☐ 7/week

b) Portion in one sitting (1 serve = 100g = 1 fillet)

☐ ½ serve ☐ 1 serve ☐ 2 serves ☐ 3 serves ☐ 4 serves ☐ 5 serves ☐ 6 serves

19. Eggs, including egg dishes such as omelette or frittata

a) Times per week

☐ Never ☐ 1/week ☐ 2/week ☐ 3/week ☐ 4/week ☐ 5/week ☐ 6/week ☐ 7/week

b) Portion in one sitting

☐ 1 egg ☐ 2 eggs ☐ 3 eggs ☐ 4 eggs ☐ 5 eggs ☐ 6 eggs

20. Cheese

a) Times per week

☐ Never ☐ 1/week ☐ 2/week ☐ 3/week ☐ 4/week ☐ 5/week ☐ 6/week ☐ 7/week

b) Portion in one sitting (1 serve = 40g or 2 slices hard cheese)

☐ ½ serve ☐ 1 serve ☐ 2 serves ☐ 3 serves ☐ 4 serves ☐ 5 serves ☐ 6 serves

21. Nuts eg loose nuts or nut spread such as peanut butter

a) Times per week

☐ Never ☐ 1/week ☐ 2/week ☐ 3/week ☐ 4/week ☐ 5/week ☐ 6/week ☐ 7/week

b) Portion in one sitting (1 serve = 1 handful or 30g or 2 tablespoons nut spread)

☐ ½ serve ☐ 1 serve ☐ 2 serves ☐ 3 serves ☐ 4 serves ☐ 5 serves ☐ 6 serves

What type of nuts?

☐ Walnuts ☐ Almonds ☐ Peanuts ☐ Cashews ☐ Pistachio ☐ Other

Do you currently take any nutritional supplements?

☐ No

☐ Yes, *please specify the type and dose*

____
